# Supplementary material for: A Humanized and Viable Animal Model for Congenital Adrenal Hyperplasia–CYP21A2-R484Q Mutant Mouse
Source: Int J Mol Sci. 2024 May 7;25(10):5062. doi: 10.3390/ijms25105062 (PMC11120801; doi:10.3390/ijms25105062)
Supplement: Supplementary file 1 [file ijms-25-05062-s001.zip › ijms-2942051-supplementary.pdf]

Supplementary figures

Supplementary figure 1

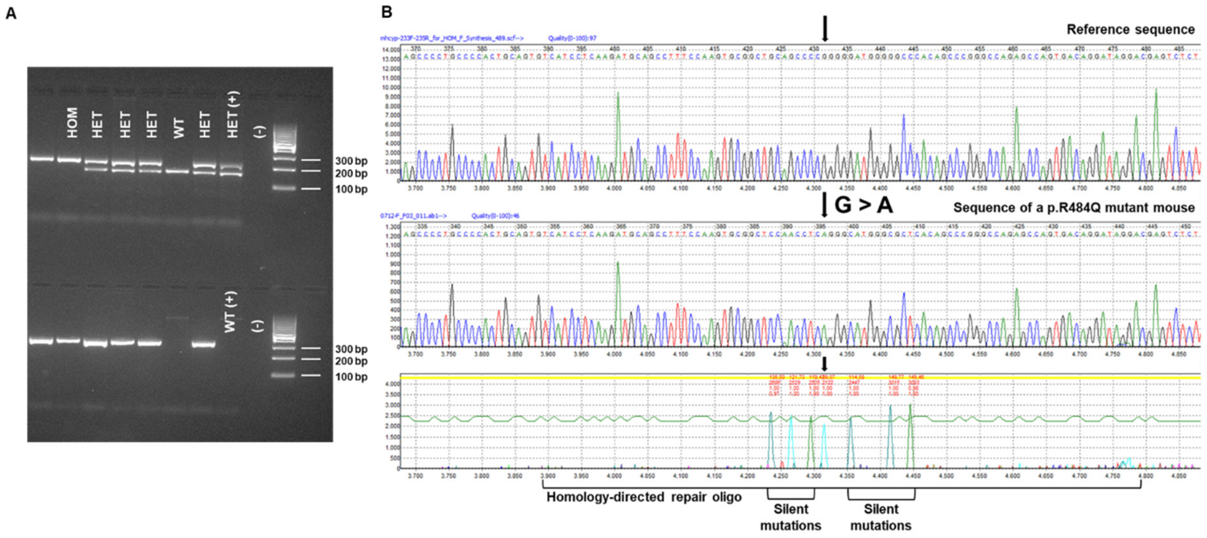

Figure S1. An example of a PCR reaction and Sanger sequencing. (A) Genotyping results of a litter and (B) Sanger sequencing of a p.R484Q mutant mouse. Genotyping results consist of two parts: TOP - Multiplex PCR reaction performed to distinguish between HOM (222 bp), HET (double band) and WT (165 bp). BOTTOM – A mutation-specific PCR reaction showing a single fragment (322 bp) for HOM and HET mice. Ladder: GeneRulerTM 100 bp DNA ladder. Sequencing chromatogram shows mutation p.R484Q represented by the change c.1451.G > A with surrounding silent mutations

Supplementary Table S1.

Table S1. Limit of detection and limit of quantification values measured during steroid hormone measurements using LC-MS/MS

No:	Steroid hormone	Limit of detection (LOD) ng/ml	Limit of quantification (LOQ) ng/ml
1	Aldosterone	0.005	0.010
2	11-Deoxycorticosterone	0.010	0.025
3	Cortisosterone	0.010	0.010
4	18OH-Cortisosterone	0.050	0.200
5	Progesterone	0.010	0.015
6	Testosterone	0.020	0.100

Supplementary figure 2.

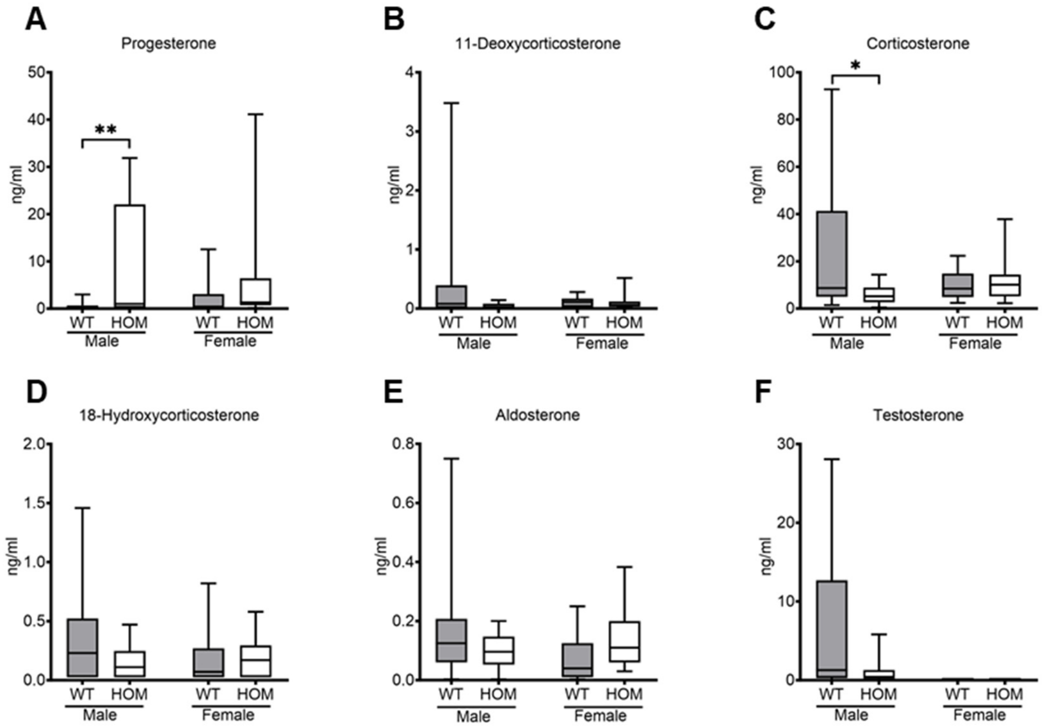

Figure S2. Steroidogenic hormone concentration levels in mutant and wildtype mice at 8-weeks. (A) Progesterone, (B) 11-Deoxycorticosterone, (C) Corticosterone, (D) 18-Hydroxycorticosterone (18-OH-corticosterone), (E) Aldosterone, (F) Testosterone levels were measured by LC-MS/MS. All values are represented in box plots with the median as a cross bar. Statistical significance was determined by Mann-Whitney test with $P < 0.05$*, $P < 0.01$**. For analysis, the plasma of 34 male mice (WT = 18; HOM = 16) and 26 female mice (WT = 13; HOM = 13) were used.

Supplementary figure 3.

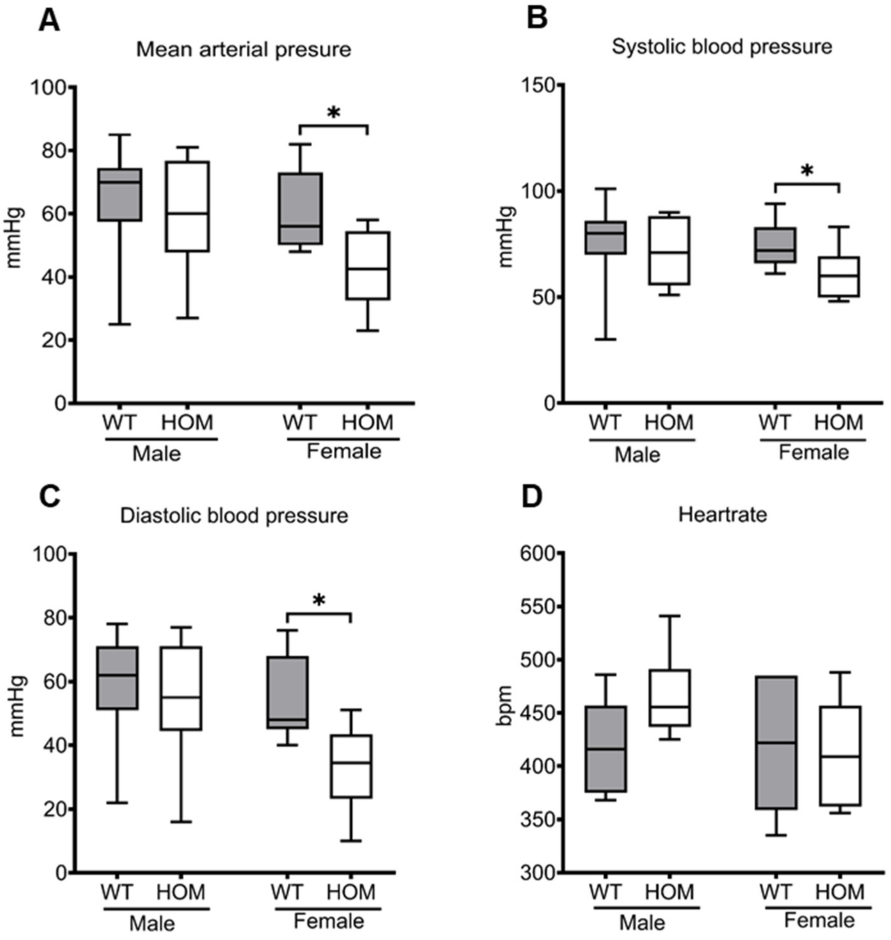

Figure S3. Blood pressure measurements in mutant and wildtype mice at 8-weeks. Blood pressure was measured three consecutive times per mouse using a non-invasive tail cuff under a constant supply of isoflurane. (A) Mean arterial pressure (MAP), (B) Systolic blood pressure (SBP), (C) Diastolic blood pressure (DBP), and (D) Heartrate. Data was processed and analysed using the Lab Chart Program. All values are represented in box plots with the median as a cross bar. Statistical significance was determined by Mann-Whitney test with $P < 0.05$*. For analysis, 19 male mice (WT = 9; HOM = 10) and 15 female mice (WT = 7; HOM = 8) were used.

Supplementary figure 4:

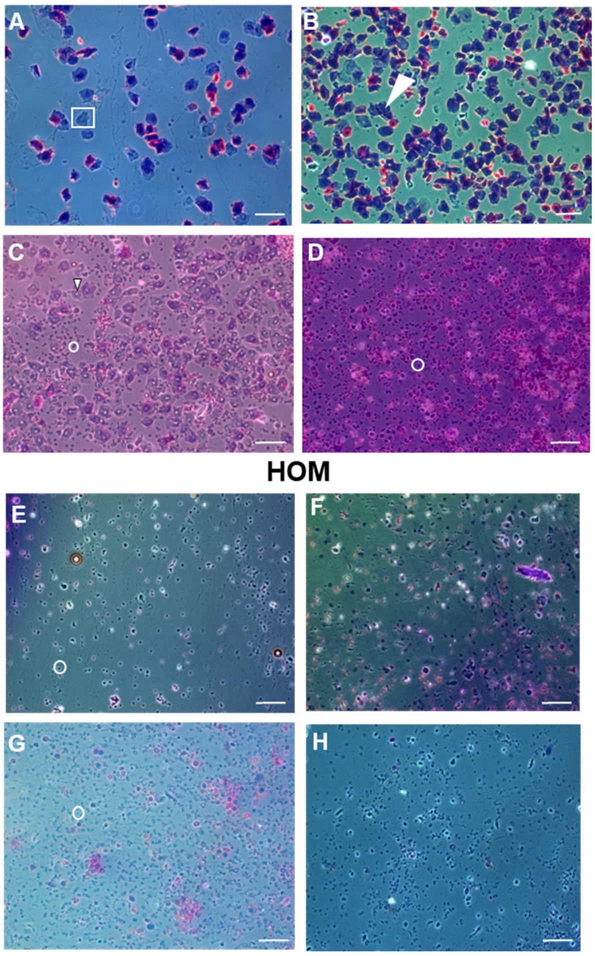

Figure S4. Vaginal cytology presenting different stages of the estrous cycle in 8-week-old mice. Three cell types were identified in vaginal smears collected using distilled water. There are three types of cells identified in the estrous cycle of a mouse – Leukocytes, nucleated epithelial cells and cornified epithelial cells. The different stages of the estrous are clearly distinguishable in the wildtype (WT) mice (A-D). The stages include (A) Proestrus – Dominated by nucleated epithelial cells (squares), (B) Estrus - Dominated by cornified epithelial cells (arrowheads), (C) Metestrus – contains all three cell types and (D) Diestrus – Dominated by leukocytes (circles). The homozygous (HOM) mice remained in a permanent diestrus phase (E-H). Scale bar: 100 µm (A-H).
